# Patients presenting with somatic complaints in general practice: depression, anxiety and somatoform disorders are frequent and associated with psychosocial stressors

**DOI:** 10.1186/1471-2296-11-67

**Published:** 2010-09-15

**Authors:** Nader Haftgoli, Bernard Favrat, François Verdon, Paul Vaucher, Thomas Bischoff, Bernard Burnand, Lilli Herzig

**Affiliations:** 1 Department of Ambulatory Care and Community Medicine, University of Lausanne, Bugnon 44, 1011 Lausanne, Switzerland; 2 Institute of General Medicine, University of Lausanne, Bugnon 44, 1011 Lausanne, Switzerland; 3 Institute of Social and Preventive Medicine, Centre Hospitalier Universitaire Vaudois and University of Lausanne, Bugnon 21, 1011 Lausanne, Switzerland

## Abstract

**Background:**

Mental disorders in primary care patients are frequently associated with physical complaints that can mask the disorder. There is insufficient knowledge concerning the role of anxiety, depression, and somatoform disorders in patients presenting with physical symptoms. Our primary objective was to determine the prevalence of depression, anxiety, and somatoform disorders among primary care patients with a physical complaint. We also investigated the relationship between cumulated psychosocial stressors and mental disorders.

**Methods:**

We conducted a multicentre cross-sectional study in twenty-one private practices and in one academic primary care centre in Western Switzerland. Randomly selected patients presenting with a spontaneous physical complaint were asked to complete the self-administered Patient Health Questionnaire (PHQ) between November 2004 and July 2005. The validated French version of the PHQ allowed the diagnosis of mental disorders (DSM-IV criteria) and the analyses of exposure to psychosocial stressors.

**Results:**

There were 917 patients exhibiting at least one physical symptom included. The rate of depression, anxiety, and somatoform disorders was 20.0% (95% confidence interval [CI] = 17.4% to 22.7%), 15.5% (95% CI = 13.2% to 18.0%), and 15.1% (95% CI = 12.8% to 17.5%), respectively. Psychosocial stressors were significantly associated with mental disorders. Patients with an accumulation of psychosocial stressors were more likely to present anxiety, depression, or somatoform disorders, with an increase of 2.2 fold (95% CI = 2.0 to 2.5) for each additional stressor.

**Conclusions:**

The investigation of mental disorders and psychosocial stressors among patients with physical complaints is relevant in primary care. Psychosocial stressors should be explored as potential epidemiological causes of mental disorders.

## Background

In primary care, physical complaints are frequently accompanied by psychological disorders and may constitute the primary, or even the only reason for the appointment with a physician[1,2]. It is reported that patients with anxiety or depression are more than twice as likely to exhibit multiple unexplained somatic symptoms as those without anxiety or depression[1,3,4]. Psychological disorders can be masked by physical complaints, such as headache, back pain, thoracic pain, or digestive troubles,[5] and under-recognition of mental disorders by a general practitioner (GP) has been frequently reported[6-8]. The absence of somatic illness was described as an important facilitator in the recognition of a mental disorder,[9] but in primary care more than half of all patients present with physical symptoms. It would appear that GPs are well placed to detect mental disorder in patients presenting with physical complaints[10]. Furthermore, GPs are able to respond to patients' expectation to explore psychosocial elements[11]. Thus, it is important to determine ways in which GPs could improve the quality of detection of mental disorders in patients with physical complaints.

Some authors have been particularly interested in clinical clues of mental disorders in Primary Care patients[12,13]. Four variables were found to be important in predicting mental disorder: recent stress, more than five somatic symptoms (with or without explained cause), poor self-reported health status, and symptom severity (S4 model)[12]. Other authors have focused specifically on medically unexplained symptoms that appear in functional somatic syndromes (chronic fatigue syndrome, fibromyalgia, irritable bowel syndrome, and non-ulcer dyspepsia) and reported an association between those syndromes and mental disorders[2,14]. Psychosocial stressors also affect the patient's state of health [15] and could also be related to mental disorders, but there have been few studies conducted regarding the possible association of mental disorders in patients presenting with physical symptoms in primary care and psychosocial stressors. [16-18]. Our objectives were to determine the prevalence of depression, anxiety, and somatoform disorders in primary care patients with a physical complaint and to explore the strength of associations between exposure to psychosocial stressors and anxiety, depression, or somatoform disorders.

## Methods

This cross-sectional study was conducted by 21 GPs and three fellow physicians in one academic primary care centre located in Western French-speaking Switzerland. Patients over 18 years of age spontaneously reporting a physical complaint at the start of the consultation were eligible whether the complaint was recurrent or not. Previous physical disorders that were not present anymore were not recorded. The inclusion of all consecutive patients with physical complaints would have interfered with the daily clinical practice, therefore each GP included one patient per each half day of consultation selected by a daily randomized identifier. We assumed that 10-12 patients would be seen per half day of consultation and that half of those patients would exhibit a somatic symptom. Therefore, we prepared a series of lists containing rank orders of eligible patients: one of the ranking numbers was randomly determined to be the eligible patient of the half-day. In the academic centre, all consecutive eligible patients were asked to participate in the study as fewer patients were eligible.

Patients were recruited from November 2004 to July 2005. Exclusion criteria were: vital emergencies, home medical consultation, phone consultation, dementia, intellectual deficiency, inability to understand French, and acute psychiatric disease preventing the patient from answering the questionnaire appropriately.

We used the French version of the full Patient Health Questionnaire (PHQ), a self-report version of the PRIME-MD, a validated procedure to identify mental disorders such as depression, anxiety, alcohol abuse, eating disorders, and somatoform disorders[19,20]. These disorders are explored through five sections of the PRIME-MD, and their interpretations are based on the DSM-IV.

As different definitions of somatoform disorders are available, we opted for the PHQ definition of multisomatoform disorder (MSD). MSD is defined by the presence of three or more bothersome unexplained physical complaints among 13 presented on a checklist, and a history of chronic somatisation[21]. MSD is detected by the PHQ questionnaire and is more appropriate for the primary care setting than the DSM IV criteria.

We used the ten psychosocial stressors defined in the 12^th ^question of the PHQ (1. worrying about own health; 2. embarrassment about weight and look; 3. little or no sexual desire nor pleasure during sex; 4. difficulties with husband/wife, partner/lover, or boyfriend/girlfriend; 5. stress due to taking care of children, parents, or other family members; 6. stress at work, outside home, or at school; 7. financial problems or worries; 8. having no one to turn to when having a problem; 9. something bad that happened recently; 10. thinking or dreaming about something terrible that happened in the past). Participants were questioned on the subjective intensity of exposure to these psychosocial stressors during the past four weeks. Major stressors are defined as those for which patients' report being "bothered a lot". Exposure and case definition were therefore defined by entirely independent questions. The PHQ was completed with socio-demographic questions. Higher education was defined as university education or equivalent.

GPs filled out another questionnaire and collected data on comorbidities, consultation length, diagnosis related to the chief complaint, and care required during the last three months. In addition, a unique identification code was used to allow GPs (and GPs only) to identify their patients easily. The main diagnoses and comorbidities were coded according to a coded list.

Patients were informed of the study and were included if they gave oral consent to participate. They explicitly acknowledged and consented to having their personal information sent to the data centre by the physician. The protocol was approved by the official Ethics Committee of the Canton of Vaud (Prot.100/04). Patients could either fill in their questionnaire at the GP's office or send it back to their GP in a sealed envelope to be transmitted to the data centre. Each GP held a patient log file, which was not transmitted to the data collection centre.

### Statistical methods

The prevalence of depression, anxiety, or somatoform disorders in enrolled patients was calculated with a 95% confidence interval (95% CI). Sample size was calculated for a significance level set at 0.05 and a precision of 2% for an expected prevalence of depression of 10%. An adequate sample size was estimated to be 864 participants. The total number of participants to be included was rounded to 1000 participants as 10% of case report forms were expected to include missing data. To explore the associations between psychosocial stressors and mental disorders using the Chi square test the significance level was set at 0.05, but multiple testing was taken into consideration using Bonferroni adjustment for 10 studied determinants. To achieve an overall significance level of 0.05, the significance level of each association was set at P < 0.005. The cumulative effect of various psychosocial stressors was then explored by calculating odds of exhibiting anxiety, depression, or somatoform disorder between patients exposed to a different number of psychosocial stressors. Patients without any stressors were chosen as the reference group. The confounding effect of age, gender, and level of education (higher versus others) was adjusted for through logistic regression. Comorbidities were recorded but were not considered as additional confounders. Data were interpreted and analyzed with StataCorp. 2008 Statistical Software: Release 10.0 (College Station, Texas: Stata Corporation). We followed the STROBE statement to prepare this report. [22].

## Results

**Figure 1:**
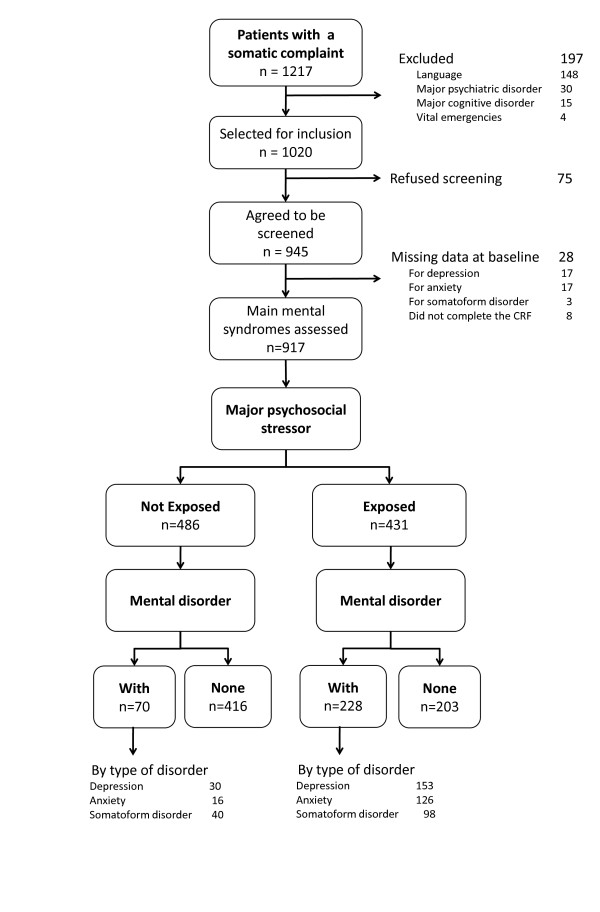
**Flow chart**.

From November 2004 to July 2005, 1020 patients were eligible for the study according to the defined criteria. A total of 917 patients were included in the analysis (90%), of which 811 were patients from a private practice and 106 were from the academic primary care centre. The 21 physicians enrolled an average of 38.2 (SD = 15.1) patients. Mean consultation length was 27 minutes (SD = 10). Details on refusals and missing data are given in Figure 1. Most patients were women (63%), the mean age was 55 years (SD = 17.6), and 78% were Swiss citizens. One-fifth of the subjects had a higher education (19%), 35% were either retired or receiving a pension, 35% were employed, 12% housewife/househusband, 4% in formation, 4% unemployed, and 10% other (i.e.: between two employments). The leading diagnostic categories related to the patient's physical complaint were musculoskeletal (30%), respiratory (12%), psychiatric (11%), digestive (9%), cardiovascular (7%), neurological (6%), infectious (5%), and other (20%). Almost two-thirds of patients had one to three consultations during the three previous months, with only 22% having no consultation during this time. Women were more likely to present somatoform disorders, and young persons or foreigners were more likely to present depression or anxiety (Table 1). Chi Square test were used to identify differences in characteristics between groups at a significant level of 0.05.

**Table 1:** Patient characteristics (N = 917)

Characteristics	All patients N = 917	Depression N = 183	Anxiety N = 142	Somatoform disorders N = 138
***Gender***				
Female	581	121 (20.8%)	102 (17.6%)	116 (20.0%)
Male	336	62 (18.4%)	40 (11.9%)	22 (6.5%)
***Age***				
< 30	94	27 (28.7%)	23 (24.5%)	19 (20.2%)
30-49	279	69 (24.7%)	57 (20.4%)	57 (20.4%)
50-69	341	60 (17.6%)	46 (13.5%)	36 (10.6%)
> = 70	203	27 (13.3%)	16 (7.9%)	26 (12.8%)
***Nationality***				
Swiss	716	130 (18.2%)	102 (14.2%)	94 (13.1%)
Other	189	53 (28.0%)	40 (21.2%)	42 (22.2%)
***Education***				
High	171	28 (16.4%)	22 (12.9%)	14 (8.2%)
Lower	716	151 (21.1%)	116 (16.2%)	117 (16.3%)
***Duration of the index consultation***				
Mean (SD) in minutes	27.4 (10.4)	30.3 (11.3)	30.7 (12.0)	29.2 (11.0)
***Number of medical consultations during the previous 3 months***				
Mean (SD)	2.1 (2.2)	3.0 (2.9)	2.8 (2.2)	2.7 (2.2)

**Figure 2:**
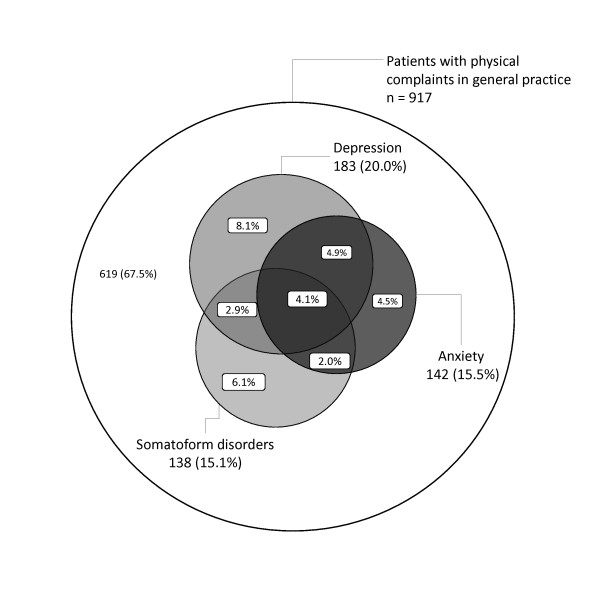
**Overlapping of depression, anxiety, and somatoform disorder for patient with a physical complaint in primary care**.

In patients with at least one physical complaint, the prevalence of depression was 20.0% (95% CI = 17.4% to 22.7%); 8.7% of these patients suffered from minor depression and 11.2% from major depression. The prevalence of anxiety was 15.5% (95% CI = 13.2% to 18.0%) and prevalence of somatoform disorders 15.0% (95% CI = 12.8% to 17.5%). An important overlapping between mental disorders was observed (Figure 2). Nearly one third (32.5%; 95% CI = 29.5% to 35.6%) of all patients presented with anxiety, depression, or somatoform disorder. Compared to the private practice, patients attending the academic primary care centre were more depressed (28.3% vs. 18.9%; p = 0.028) and were more often anxious (21.7% vs. 14.7%; p = 0.064).

**Figure 3:**
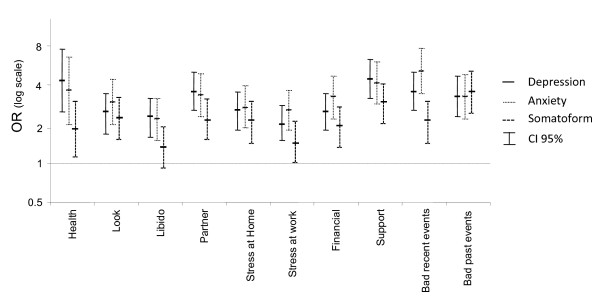
**Odds of having depression, anxiety, or somatoform disorder for patients exposed to different psychosocial stressors**.

All ten studied psychosocial stressors were significantly (p < 0.005) associated with depression and anxiety. For somatoform disorders, only eight stressors were significantly associated (libido and stress at work were not significantly associated) (Figure 3). We also observed an increased prevalence of mental disorder along with a higher level of exposure (not bothered at all, bothered a little, bothered a lot) to each psychosocial stressor addressed in the PHQ. All psychosocial stressors were similarly associated with depression, anxiety, and somatoform disorders. Exposure to up to five concurrent major psychosocial stressors was related to an increasing prevalence of all three studied mental disorders. Only 14.4% of patients without any major stressor had mental disorder, whereas over 90% of those exposed to five or more major stressors presented at least one of the three mental disorders (Table 2). Furthermore, each major psychosocial stressor increased the association to mental disorders by 2.2 times (95% CI = 2.0 to 2.5). The likelihood-ratio test shows that the cumulating effect of a psychosocial stressor can be assumed to be linear (p = 0.568). The associations were not confounded by age, gender, or education level. In our cohort, the strength of the association between psychosocial stressors and depression, anxiety, and somatoform disorders was much more important than with any observed socio-demographic determinant (level of education, occupation, marital status, nationality, or living alone).

**Table 2:** Strength of the association between depression, anxiety, or somatoform disorders and the number of major psychosocial stressors

Number of major psychosocial stressors	Prevalence of depression, anxiety or somatoform disorders n/N (%)	Odds Ratio (unadjusted) OR	OR adjusted for age, gender and level of education OR_ADJ _(CI95%)
***None***	70/486 (14.4%)	1.0	1.0
***1***	57/168 (33.9%)	3.1	3.2 (CI95% 2.1; 4.8)
***2***	54/114 (47.4%)	5.3	5.0 (CI95% 3.1; 7.9)
***3***	35/56 (62.5%)	9.9	10.3 (CI95% 5.4; 19.3)
***4***	29/37 (78.4%)	21.5	21.5 (CI95% 9.2; 50.4)
***5***	23/25 (92.0%)	68.3	74.7 (CI95% 16.9; 330.1)
***≥6***	30/31 (96.8%)	178.3	207 (CI95% 27.2; 1573)

## Discussion

In this study, we found a substantial prevalence of depression (20.0%), anxiety (15.5%), and somatoform disorders (15.1%) in patients consulting a GP for at least one physical complaint. All psychosocial stressors defined by Spitzer et al. [20] were clearly associated with depression, anxiety, and somatoform disorders. Stressors appear to have a slightly lower impact on levels of somatoform disorders than on anxiety and depression. Our study does not allow explaining this difference, but we suppose that patients with anxiety or depression may have different psychological mechanism than patients with somatoform disorder to cope with these stressors. The prevalence of mental disorders increased with the subjective perception of higher intensity exposure to these stressors, i.e. if they were endorsed as "bothered a lot".

The strengths of this study are the large sample size, the number and diversity of participating practitioners, and the use of standardized, distinct, validated measurements to determine mental disorders and psychosocial stressors. The inclusion of a large number of participants increased the precision of our measurements. Selecting patients using randomized lists and recruiting subjects from a large number of GPs in a variety of settings decreased the risk of selection bias. Our results are therefore believed to be relevant for most patients in primary care in western Switzerland even if our mean consultation length of 27 minutes seems to be longer than usual encounters [23]. In Switzerland the mean consultation length is however of 15.6 minutes, and is longer than in other European countries. Moreover, filling-up of questionnaires and complementary psychosocial questions addressed lengthened the consultation. We relied on the PHQ, a standardized, validated translated questionnaire on mental and psychosocial disorders. Finally, distinguishing depression from anxiety and somatic disorders allows for a better estimate of the impact of these disorders on a physician's workload.

Our study is limited by its design, case definition, and setting. The comparison between our results of prevalence and those of other studies must be made with caution due to the different definitions used. Care must be taken in considering questionnaire data as a valid method for detecting mental syndromes. The PHQ is not the best design to discern mental disorder and psychosocial stressors; a structured interview by an external psychologist would have been a preferred gold standard. Furthermore, no consensus exists in defining a gold standard for somatoform disorders. Our case definition was based on multisomatoform disorder as defined by Kroenke [21] and might not be equivalent to those chosen in other studies. In addition, our results cannot be generalized to the entire population as they were based on a convenience sample of primary care practices, and limited to patients with a physical complaint in primary care, albeit intentionally. Furthermore, this cross-sectional study was not designed to observe causal relationships between psychosocial stressors and mental disorders. Some factors, such as a sexual disorder, are known to be a consequence of mental disorder. Further studies are needed to assess this causal association. Finally, we did not record previous exposition to psychosocial factors either making it difficult to know if the potential confounding effect of past physical disorders were not also related to psychosocial stressors. The study design does not make it possible to test sequence of events and therefore does not try to adjust for such potential confounding effects.

Our results are similar to those observed in four primary care clinics in the framework of the original PRIME-MD study, from which the PHQ questionnaire was derived[19]. Spitzer et al. found similar figures for prevalence of major depression (7 to 19%), minor depression (2 to 9%), anxiety disorder (10 to 25%), and somatoform disorders (9 to 29%). Similar results were also observed in the study validating the PHQ[20]. Both of these studies revealed important variation among different primary care centers, suggesting that a patient's profile might be influenced by the location and type of service offered by a clinical center. Using structured interviews by psychiatrists, Pini et al. also found a prevalence of depression of 15.6%. Munk-Jorgensen et al. [24] found a lower prevalence of anxiety in Scandinavian general practice than we did (4.8% for men and 6% for women). However, they included all patients, while we included only patients with physical complaints, which could explain the notable difference between our observations and similar studies in general practice[25]. Finally, 21.9% of primary care patients in a Dutch study had somatoform disorders[26]. Löwe et al.[27] reported an overlap between depression, anxiety and somatoform disorder, but with much lower prevalence than our findings. This could be explained by the fact that they reported severe levels of depression, anxiety or somatoform disorder and included patients with our without physical complaints.

Prevalence of mental disorders differs among age groups. In the general population, older people have a higher risk of presenting with mental disorders, whereas in primary care, younger patients are at a higher risk[2,28]. One reason for the lower prevalence of depression, anxiety, and somatoform disorders in older patients may be that physical complaint is more often associated with a somatic problem in older rather than younger patients. The higher prevalence of mental disorders in women is described in the literature [29] but was not clearly notable in our cohort for depression.

Compared to usual socio-demographic measurements, the subjective perception of psychosocial stress exhibited a much stronger association with the presence of anxiety, depression, and somatoform disorder, suggesting that subjective major psychosocial stressors are clearly related to the most frequent mental disorders. Moreover, we observed that the number of psychosocial stressors had a cumulative effect on the prevalence of anxiety or depression, and could be a powerful marker for mental disorders. However, our study does not make it possible to identify the time sequence of events. Reverse causality cannot be excluded. In our review of the literature, we found little epidemiological data regarding the association between psychosocial stressors and these disorders[30]. In primary care in particular there is evidence that investigating psychosocial factors could potentially be helpful. Badger et al. determined that investigating these factors improves a GP's ability to detect depression[31]. Moreover, Frietzsche et al. found that 79% of patients considered the investigation of psychosocial aspects to be important, and two thirds of patients believed that it could have a healing effect[11].

## Conclusions

While the relationship between somatic, depressive, and anxiety symptoms is well-established, the relationship between all three types of symptoms and psychosocial stressors remained questionable. Patients cumulating psychosocial stressors are more likely to present mental disorders than others. The relationship is dose dependant and therefore supports the causal explanation between social distress and depression, anxiety and somatoform disorders. GPs could play an important role in exploring and discussing patients' mental state and their psychosocial environment. Our study showed that investigating these factors is relevant to the GP's daily practice. Studies are needed to investigate the potential benefits of an integrated strategy of caring for psychosocial stressors to help improve somatic complaints and to further investigate the eventual causal relationship to mental disorders.

## Ethical approval, and competing interests

The study protocol was approved by the official State Ethics Committee of the Canton of Vaud (Prot.100/04). The authors have no competing interests.

## Abbreviations

CI: confidence interval; GP: general practitioner; MSD: multisomatoform disorder; OR: odds ratio; PHQ: patient health questionnaire; SD: standard deviation

## Authors' contributions

NH participated in the patients inclusion, in the preparation of study data and drafted the manuscript; BF participated in the design of the study and in the draft of the manuscript; FV participated in the design of the study and in the patients inclusion; PV performed the statistical analysis and helped to draft the manuscript; TB participated in the design of the study and in the patients inclusion; BB participated in the design of the study and in the draft of the manuscript; LH conceived the study, was responsible of its design and coordination, participated in the patients inclusion and in the draft of the manuscript. All authors accepted the manuscript after lecture.

## Pre-publication history

The pre-publication history for this paper can be accessed here:

http://www.biomedcentral.com/1471-2296/11/67/prepub

## References

[B1] Katon W Sullivan M Walker E Medical symptoms without identified pathology: relationship to psychiatric disorders, childhood and adult trauma, and personality traits Ann Intern Med 2001 134 9 Pt 2 917 925 11346329 10.7326/0003-4819-134-9_part_2-200105011-00017

[B2] Haug TT Mykletun A Dahl AA The association between anxiety, depression, and somatic symptoms in a large population: the HUNT-II study Psychosom Med 2004 66 6 845 851 10.1097/01.psy.0000145823.85658.0c 15564348

[B3] Simon GE VonKorff M Piccinelli M Fullerton C Ormel J An international study of the relation between somatic symptoms and depression N Engl J Med 1999 341 18 1329 1335 10.1056/NEJM199910283411801 10536124

[B4] Kroenke K Spitzer RL Williams JB Linzer M Hahn SR deGruy FVrd Brody D Physical symptoms in primary care. Predictors of psychiatric disorders and functional impairment Arch Fam Med 1994 3 9 774 779 10.1001/archfami.3.9.774 7987511

[B5] Kroenke K Spitzer RL deGruy FV Swindle R A symptom checklist to screen for somatoform disorders in primary care Psychosomatics 1998 39 3 263 272 9664773 10.1016/S0033-3182(98)71343-X

[B6] Menchetti M Murri MB Bertakis K Bortolotti B Berardi D Recognition and treatment of depression in primary care: effect of patients' presentation and frequency of consultation J Psychosom Res 2009 66 4 335 341 10.1016/j.jpsychores.2008.10.008 19302892

[B7] Schulberg HC Burns BJ Mental disorders in primary care: epidemiologic, diagnostic, and treatment research directions Gen Hosp Psychiatry 1988 10 2 79 87 10.1016/0163-8343(88)90092-8 3282988

[B8] Weich S Lewis G Donmall R Mann A Somatic presentation of psychiatric morbidity in general practice Br J Gen Pract 1995 45 392 143 147 7772392 PMC1239175

[B9] Furedi J Rozsa S Zambori J Szadoczky E The role of symptoms in the recognition of mental health disorders in primary care Psychosomatics 2003 44 5 402 406 10.1176/appi.psy.44.5.402 12954914

[B10] Lowe B Grafe K Kroenke K Zipfel S Quenter A Wild B Fiehn C Herzog W Predictors of psychiatric comorbidity in medical outpatients Psychosom Med 2003 65 5 764 770 10.1097/01.PSY.0000079379.39918.17 14508018

[B11] Fritzsche K Armbruster U Hartmann A Wirsching M Psychosocial primary care - what patients expect from their General Practitioners A cross-sectional trial BMC Psychiatry 2002 2 5 10.1186/1471-244X-2-5 12000687 PMC113265

[B12] Jackson JL O'Malley PG Kroenke K Clinical predictors of mental disorders among medical outpatients. Validation of the "S4" model Psychosomatics 1998 39 5 431 436 9775700 10.1016/S0033-3182(98)71302-7

[B13] Kroenke K Jackson JL Chamberlin J Depressive and anxiety disorders in patients presenting with physical complaints: clinical predictors and outcome Am J Med 1997 103 5 339 347 10.1016/S0002-9343(97)00241-6 9375700

[B14] Henningsen P Zimmermann T Sattel H Medically unexplained physical symptoms, anxiety, and depression: a meta-analytic review Psychosom Med 2003 65 4 528 533 10.1097/01.PSY.0000075977.90337.E7 12883101

[B15] Singh-Manoux A Adler NE Marmot MG Subjective social status: its determinants and its association with measures of ill-health in the Whitehall II study Soc Sci Med 2003 56 6 1321 1333 10.1016/S0277-9536(02)00131-4 12600368

[B16] Barnow S Linden M Lucht M Freyberger H-J The importance of psychosocial factors, gender, and severity of depression in distinguishing between adjustment and depressive disorders Journal of Affective Disorders 2002 72 1 71 78 10.1016/S0165-0327(01)00424-4 12204319

[B17] Van Houdenhove B Psychosocial stress and chronic pain European Journal of Pain 2000 4 3 225 228 10.1053/eujp.2000.0189 10985865

[B18] Poleshuck EL Bair MJ Kroenke K Damush TM Tu WZ Wu JW Krebs EE Giles DE Psychosocial stress and anxiety in musculoskeletal pain patients with and without depression General Hospital Psychiatry 2009 31 2 116 122 10.1016/j.genhosppsych.2008.10.003 19269531 PMC2677657

[B19] Spitzer RL Williams JB Kroenke K Linzer M deGruy FV Hahn SR Brody D Johnson JG Utility of a new procedure for diagnosing mental disorders in primary care. The PRIME-MD 1000 study JAMA 1994 272 22 1749 1756 10.1001/jama.272.22.1749 7966923

[B20] Spitzer RL Kroenke K Williams JB Validation and utility of a self-report version of PRIME-MD: the PHQ primary care study. Primary Care Evaluation of Mental Disorders. Patient Health Questionnaire JAMA 1999 282 18 1737 1744 10.1001/jama.282.18.1737 10568646

[B21] Kroenke K Spitzer RL deGruy FV Hahn SR Linzer M Williams JB Brody D Davies M Multisomatoform disorder. An alternative to undifferentiated somatoform disorder for the somatizing patient in primary care Arch Gen Psychiatry 1997 54 4 352 358 9107152 10.1001/archpsyc.1997.01830160080011

[B22] von Elm E Altman DG Egger M Pocock SJ Gotzsche PC Vandenbroucke JP The Strengthening the Reporting of Observational Studies in Epidemiology (STROBE) statement: guidelines for reporting observational studies J Clin Epidemiol 2008 61 4 344 349 10.1016/j.jclinepi.2007.11.008 18313558

[B23] Deveugele M Derese A van den Brink-Muinen A Bensing J De Maeseneer J Consultation length in general practice: cross sectional study in six European countries BMJ 2002 325 7362 472 10.1136/bmj.325.7362.472 12202329 PMC119444

[B24] Munk-Jorgensen P Allgulander C Dahl AA Foldager L Holm M Rasmussen I Virta A Huuhtanen MT Wittchen HU Prevalence of generalized anxiety disorder in general practice in Denmark, Finland, Norway, and Sweden Psychiatr Serv 2006 57 12 1738 1744 10.1176/appi.ps.57.12.1738 17158488

[B25] Hanel G Henningsen P Herzog W Sauer N Schaefert R Szecsenyi J Löwe B Depression, anxiety, and somatoform disorders: Vague or distinct categories in primary care? Results from a large cross-sectional study Journal of Psychosomatic Research 2009 67 3 189 197 10.1016/j.jpsychores.2009.04.013 19686874

[B26] de Waal MW Arnold IA Eekhof JA van Hemert AM Somatoform disorders in general practice: prevalence, functional impairment and comorbidity with anxiety and depressive disorders Br J Psychiatry 2004 184 470 476 10.1192/bjp.184.6.470 15172939

[B27] Lowe B Spitzer RL Williams JB Mussell M Schellberg D Kroenke K Depression, anxiety and somatization in primary care: syndrome overlap and functional impairment Gen Hosp Psychiatry 2008 30 3 191 199 10.1016/j.genhosppsych.2008.01.001 18433651

[B28] Klapow J Kroenke K Horton T Schmidt S Spitzer R Williams JB Psychological disorders and distress in older primary care patients: a comparison of older and younger samples Psychosom Med 2002 64 4 635 643 10.1097/01.PSY.0000021942.35402.C3 12140354

[B29] Gorman JM Gender differences in depression and response to psychotropic medication Gend Med 2006 3 2 93 109 10.1016/S1550-8579(06)80199-3 16860269

[B30] Villaverde Ruiz M Gracia Marco R Morera Fumero A Relacion entre el estres psicosocial y la patologia psiquica: un estudio comunitario Actas Esp Psiquiatr 2000 28 1 1 5 10758422

[B31] Badger LW deGruy FV Hartman J Plant MA Leeper J Anderson R Ficken R Gaskins S Maxwell A Rand E Patient presentation, interview content, and the detection of depression by primary care physicians Psychosom Med 1994 56 2 128 135 8008799 10.1097/00006842-199403000-00008

